# Face Mask Wearing Behaviors, Depressive Symptoms, and Health Beliefs Among Older People During the COVID-19 Pandemic

**DOI:** 10.3389/fmed.2021.590936

**Published:** 2021-02-05

**Authors:** Rick Yiu Cho Kwan, Paul Hong Lee, Daphne Sze Ki Cheung, Simon Ching Lam

**Affiliations:** ^1^School of Nursing, The Hong Kong Polytechnic University, Kowloon, Hong Kong; ^2^Centre for Gerontological Nursing, School of Nursing, The Hong Kong Polytechnic University, Kowloon, Hong Kong; ^3^The Squina International Centre for Infection Control, School of Nursing, The Hong Kong Polytechnic University, Kowloon, Hong Kong

**Keywords:** COVID-19, older people, health beliefs, depressive symptoms, face mask wearing behaviors

## Abstract

The COVID-19 pandemic has affected more than 100 countries. Despite the global shortage of face masks, the public has adopted universal mask wearing as a preventive measure in many Asian countries. The COVID-19 mortality rate is higher among older people, who may find that wearing a face mask protects their physical health but jeopardizes their mental health. This study aimed to explore the associations between depressive symptoms, health beliefs, and face mask wearing behaviors among older people. By means of an online survey conducted between March and April 2020, we assessed depressive symptoms, health beliefs regarding COVID-19, and face mask use and reuse among community-dwelling older people. General linear models were employed to explore the associations among these variables. Of the 355 valid participants, 25.6% experienced depressive symptoms. Health beliefs regarding the perceived severity of disease (*p* = 0.001) and perceived efficacy of practicing preventive measures (*p* = 0.005) were positively associated with face mask use. Those who reused face masks (*p* = 0.008) had a stronger belief in disease severity (*p* < 0.001), had poorer cues to preventive measures (*p* = 0.002), and were more likely to experience depressive symptoms. Mask reuse was significantly associated with depression only among those who perceived the disease as serious (*p* = 0.025) and those who had poorer cues to preventive measures (*p* = 0.004). In conclusion, health beliefs regarding perceived severity and efficacy contributed to more frequent face mask use, which was unrelated to depressive symptoms. Older people who had a stronger belief in disease severity had less adequate cues to preventive measures and reused face masks experienced greater depressive symptoms. A moderation effect of health beliefs (i.e., disease severity and cues to preventive measures) on face mask reuse and depression was observed.

## Introduction

The Coronavirus Disease 2019 (COVID-19) pandemic is a global health issue, with more than 42 million people infected globally and 1.1 million deaths as of October 25, 2020 (1). The mortality rate from COVID-19 is higher among older people (2). While the general population in Hong Kong is highly compliant with universal mask wearing (3), there have been three waves of outbreaks with a total of 5,285 confirmed cases and 105 deaths as of October 24, 2020 (4). Despite Hong Kong's status as an international travel hub and its proximity to Wuhan, China, compliance with infection control measures among the general public has helped reduce the magnitude of the outbreak (5, 6). However, compared to people aged below 55–65 years, older people have been found to have lower levels of health literacy as concluded in a systematic review (odds ratio = 4.20) (7). Not knowing how to take effective preventive measures against COVID-19 further increases the vulnerability of this demographic group.

During the COVID-19 pandemic, as reported in a cross-sectional study conducted from February 14 to March 2, 2020 on participants recruited from outpatient departments and health centers in Vietnam (*N* = 4,029) (8), depressive symptoms have been reported more often in people aged 60 and older than in younger people aged between 18 and 39 years (odds ratio = 2.69) because of (a) pandemic-specific risk factors such as unforeseeable threats (9), social isolation, and personal and economic burden (3), (b) pre-pandemic risk factors, such as social crises in some regions, and unmet mental health services (10), and (c) age-specific risk factors, such as comorbidity of brain disorders or systemic diseases, psychosocial changes including bereavement and loss, change of role and loss of social status, and receiving institutional care (11, 12). This higher rate of depressive symptoms may also be due to the implementation of strict quarantine measures, which have kept a large number of people in isolation and affected many aspects of people's lives (13). The problems of depressive symptoms among older people are multidimensional and more complicated than in other age groups. Managing the mental health issues of older people is a priority during the COVID-19 pandemic (14). In addition to being a primary health interest, depressive symptoms are also known to have a significant effect on health behaviors (15).

The use of face masks is thought to be important in preventing respiratory infection during the pandemic because it can prevent both the acquisition and transmission of pathogens (16). On June 5, 2020, the World Health Organization (WHO) advised governments to advocate face mask use for the general public under certain conditions only, such as in areas with known or suspected community transmission, settings where physical distancing is not possible, and among people with any symptoms suggestive of COVID-19 (17). However, the evidence shows that COVID-19 can be transmitted by asymptomatic carriers (18). Some experts have recommended that masks should be worn following the precautionary principle that we should sometimes act without definitive evidence, and that even limited protection could prevent some transmission of COVID-19 and save lives (19). In some places in Asia (e.g., the Republic of Korea and Hong Kong), masks are universally worn by the public (20). It is common for those not wearing masks in public in some Asian countries to experience social shaming (21). Given the global shortage of masks during the pandemic (9), there is no option for some but to reuse face masks. However, despite the reuse of face masks (54% from a total sample of 11,072) being widely reported in Hong Kong (3), little is known about whether reusing face masks (without reuse guidelines and safety protocols) when they are in short supply is associated with depressive symptoms. Other infection control measures such as hand hygiene, personal hygiene, environmental hygiene, and social distancing have been implemented since the outbreak (22). We found no specific recommendations or preventive measures dedicated solely to the community-dwelling older people in Hong Kong, except those in residential care homes where policies have been implemented to limit visits, to establish a COVID-19-targeted group testing scheme, and to avoid non-essential travel (23).

The Health Belief Model (HBM) is one of the most widely used conceptual frameworks in health behavior research (24). The HBM is used to explain changes in and maintenance of health behaviors. The model explains that people take action to prevent illness-causing conditions because of several factors: susceptibility, severity, cues to preventive measures, benefits and barriers to a specific behavior, and self-efficacy (24). In the specific context of the COVID-19 pandemic, several studies have shown that people's beliefs regarding contracting a disease (COVID-19) and practicing preventive behaviors (e.g., face mask use) interact with their mental health (e.g., depressive symptoms) (3, 25, 26). From these studies involving different samples, it was found that the COVID-19-related health beliefs and infection control behaviors triggered by the pandemic would escalate pre-pandemic depressive symptoms and initiate pandemic-specific mental health problems. The triggering reasons commonly included feeling overwhelmed by the perceived risk of COVID-19 infection, being uncertain of the risk of infection, and feeling ill-equipped to protect themselves (3, 9, 25). Subsequently, the resulting cognitive distortions regarding high infection susceptibility, perceived poor self-efficacy, and/or negative outcome expectations associated with depressive symptoms would further strengthen and prolong the depressive symptoms.

Although these observations pertained to different populations, they were anticipated to be similar in older populations. Preventive behaviors caused by a group of factors related to health beliefs (24) play a key role in preventing older people from contracting COVID-19. These factors include susceptibility to COVID-19, the severity of the consequences of COVID-19, cues to action on the implementation of preventive measures, knowledge about COVID-19, and the efficacy of implementing preventive measures. However, there is a lack of understanding of how health beliefs are associated with face mask wearing behavior. Previous studies have also shown that health beliefs predict depressive symptoms in older people with diabetes (27). There is also a lack of understanding of how health beliefs are associated with depressive symptoms during this pandemic period. Therefore, we conducted a cross-sectional study to investigate the prevalence of depressive symptoms among the older population. Our objectives were guided by the following five research questions:

1. Are depressive symptoms associated with face mask use?
2. Are health beliefs associated with face mask use?
3. Is the reuse of face mask associated with depressive symptoms?
4. Are health beliefs associated with depressive symptoms?
5. Do health beliefs have a moderating effect on the reuse of face masks and depressive symptoms?

## Materials and Methods

### Study Design and Settings

This study employed a cross-sectional observational design and was conducted from March to April 2020 in community-dwelling older people in Hong Kong.

### Samples and Sample Size

Convenience samples were recruited using online platforms, including personal and organizational Facebook pages, a discussion forum, and a peer support group. Subjects fulfilling the following eligibility criteria were included in the study: (1) age ≥ 60 years and (2) community-dwelling for the previous 6 months. A total sample of 369 participants was estimated based on the Cochran formula for the sampling size calculation $N=\frac{Z^{2}p\left(1-p\right)}{d^{2}}$ (28), where *Z* = 1.96 for the 95% confidence level, *p* = 40% for the estimated prevalence of depressive symptoms in the general public (29), and *d* = 5% for an acceptable margin of error (28).

### Variables and Measurement

To describe the characteristics of the subjects, data on their age, sex, educational level, number of household members, and marital status were collected as demographic variables. The three key variables in this study were depressive symptoms, health beliefs, and the wearing of face masks, including face mask use and face mask reuse practices.

#### Depressive Symptoms

The Patient Health Questionnaire−9 (PHQ9) was employed to measure depressive symptoms (30). PHQ9 includes nine items measured on a 4-point frequency scale, with total scores ranging from 0 to 27. A higher score indicates a higher level of depressive symptomatology. The Chinese version of the PHQ9 was validated by comparing its scores with the clinical diagnosis of a major depressive episode using the DSM-IV criteria (AUC = 0.95, sensitivity = 0.88, specificity = 0.88) at the cut-off point of 9/10 with good internal consistency (Cronbach's α = 0.89) (30). The PHQ9 was validated among Chinese older people aged equal to or over 60 years and was found to show good validity (sensitivity = 0.86, specificity = 0.77) for identifying major depression in late life at the cut-off point of 9/10 (31).

#### Health Beliefs

Health beliefs pertaining to actions to prevent infection were measured using an instrument developed by our team based on the Health Belief Model and modified from the scales used during the 2003 Severe Acute Respiratory Syndrome (SARS) epidemic in Hong Kong (24, 32). The health belief instrument is comprised of 13 items rated using 4-point frequency scales (11 items) and dichotomous scales (2 items). The instrument consists of five constructs to measure five domains of health-related beliefs following the Health Belief Model. The first construct is the perceived susceptibility to current infectious outbreaks (3 items, possible range 1–6): a higher score indicates a stronger belief in the likelihood of getting the disease. Second, perceived severity of current infectious outbreak (2 items, possible range 2–8) is where a higher score indicates a stronger belief in disease severity and its sequelae. Third, cues-to-action for preventive measures (5 items, possible range 5–20) is where a higher score indicates having more strategies to activate measures to prevent against the disease. The fourth construct is the person's knowledge of current infectious outbreak (2 items, possible range 2–8): a higher score indicates a stronger belief in efficacy of the advised action to reduce risk or seriousness of impact and about the tangible and psychological costs of the advised action. Fifth, the efficacy to practice preventive measures (1 item, possible range 1–4) is the last construct, where a higher score indicates a higher level of confidence in one's ability to respond against the disease.

#### Face Mask Wearing Behaviors

The Face Mask Use Scale (FMUS) was employed to measure the practice of face mask use (33). The FMUS is comprised of six items measuring how frequently a person wears a mask in the following situations: (1) in public venues for protection against respiratory infection, (2) in a doctor's clinic for protection against respiratory infection, (3) at home when the person has symptoms of respiratory infection, (4) in public venues when the person has symptoms of respiratory infection, (5) in a doctor's clinic when the person has symptoms of respiratory infection, and (6) at home when family members have a respiratory infection. Frequency was measured on a 5-point scale (1 = *never*, 2 = *rarely*, 3 = *sometimes*, 4 = *frequently*, and 5 = *always*), with a total score ranging from 6 to 30, where a higher score indicates more frequent face mask use. The FMUS has been validated and shows good internal consistency (Cronbach's α = 0.81) and test-retest reliability (ICC = 0.84) (33).

Face mask reuse was measured using a single-item question, “During the current outbreak, how often do you reuse a face mask?” with a 5-level frequency response and analyzed in a dichotomous manner (No = *never reuse/rarely* [i.e., reuse 0–2 times], Yes = *reuse more than twice*).

### Statistical Methods

The distribution of subject variables (i.e., demographics and clinical outcomes) was described using means (SD) and frequency (%) according to the levels of measurement of the variables. Descriptive statistics were also used to report the prevalence of depressive symptoms among the older population. The prevalence of depressive symptoms was estimated from the participants' screening PHQ-9 scores (using a cut-off score above 9 to define a positive case) with a 95% confidence interval. Considering that the population of community-dwelling older people in Hong Kong is 1.07 million (34), general linear models were employed to examine the five objectives formulated in the Introduction. For objective #1, the dependent variable was participants' face mask use, indicated by scores on the FMUS, and the independent variable was depressive symptoms. For objective #2, the dependent variable was participants' face mask use and the independent variable was health beliefs. For objective #3, the dependent variable was depressive symptoms and the independent variable was face mask reuse. For objective #4, the dependent variable was depressive symptoms and the independent variable was health beliefs. For objective #5, the subjects were divided into two groups to test the moderating effect of health beliefs. Health belief domains significantly associated with depressive symptoms were selected to test the moderating effect. The high health belief group included subjects with a health belief score equal to or above the median, while the low health belief group included subjects with a health belief score below the median. The dependent variable was depressive symptoms, and the independent variable was face mask reuse. The moderating effect was supported by a subgroup analysis if face mask reuse was significantly associated with depressive symptoms in one health belief sub-group but not the other. All models were adjusted for age, sex, education level, and number of members in the household. The level of significance was set at 0.05.

### Ethics

Written consent was obtained from all participants. The study was approved by the Human Subjects Ethics Sub-committee of the Hong Kong Polytechnic University (Reference number: HSEARS20200227002-01). Subjects were provided with access to a medical service hotline to seek medical support if they experienced mood disturbances (e.g., depressive mood) after completion of the online survey.

## Results

Of the 370 recruited subjects, data from 15 (4.0%) were disqualified from data analysis either because of incomplete data (*n* = 9) or acquiescence bias (i.e., identified as a “yea-sayer” or “nay-sayer” on 90% of the items, *n* = 6). A total of 355 subjects completed the online surveys. As shown in Table 1, their mean age was 62.85 (SD: 3.30) years (range: 60–75). Two-thirds of the participants were female (*n* = 241, 67.9%), and more than half were university graduates (*n* = 196, 55.2%). The mean number of persons in the household was 2.42 (SD: 1.45). Most of them were married (*n* = 250, 70.4%), and the prevalence of depression was 25.6% (95%CI: 23.3–27.9). The mean score for depressive symptoms as measured by the PHQ9 was 6.59 (SD: 5.74), for health beliefs was 32.88 (SD: 3.03), and for face mask use (i.e., FMUS) was 25.08 (SD: 4.68). Most participants did not report reusing face masks (*n* = 294, 82.8%). There were missing data for the sex variable (*n* = 3, 0.9%) and number of household members (*n* = 1, 0.3%). There were no missing data for any of the other variables.

**Table 1:** Demographics and clinical outcomes.

	***N* = 355 Mean ± SD/frequency (%)**
**Demographics**	
Age (range: 60–75 years)	62.85 ± 3.30
**Gender**	
Male	111 (31.3)
Female	241 (67.9)
Missing	3 (0.9)
**Education**	
Elementary or below	12 (3.4)
High school	147 (41.4)
University or higher	196 (55.2)
No. of household members (range: 0–10 people)	2.42 ± 1.45
Missing	1 (0.3)
**Marital status**	
Single	54 (15.2)
Married	250 (70.4)
Divorced/Separated	31 (8.7)
Widowed	20 (5.6)
**Clinical outcomes**	
Depression (PHQ9 cut-off: 9/10)	
Yes	91 (25.6)
No	264 (74.4)
Depressive symptoms, PHQ9 (possible range: 0–27)	6.59 ± 5.74
Health beliefs (possible range: 11–46)	32.88 ± 3.03
Susceptibility (possible range: 1–6)	2.90 ± 0.96
Severity (possible range: 2–8)	5.98 ± 1.53
Cue to action (possible range: 5–20)	14.78 ± 1.78
Knowledge (possible range: 2–8)	5.73 ± 1.16
Efficacy (possible range: 1–4)	3.46 ± 0.60
Facemask Use Scale, FMUS (possible range: 6–30)	25.08 ± 4.68
In public venues to protect myself (possible range: 1–5)	4.75 ± 0.72
In doctor's clinic to protect myself (possible range: 1–5)	4.80 ± 0.73
At home when I have symptoms (possible range: 1–5)	3.23 ± 1.62
In public venues when I have symptoms (possible range: 1–5)	4.59 ± 0.98
In doctor's clinic when I have symptoms (possible range: 1–5)	4.79 ± 0.76
At home when family members have symptoms (possible range: 1–5)	2.91 ± 1.65
**Face mask reuse**	
Yes (more than twice)	61 (17.2)
No (never/rarely, < two times)	294 (82.8)

*PHQ9, Patient Health Questionaire-9*.

For objective #1, Model 1 in Table 2 showed that depressive symptoms were not associated with face mask use after adjusting for age, sex, education, and number of members of the household. For objective #2, results revealed that two health belief components were observed to be positively associated with face mask use, namely severity (β = 0.63, *p* = 0.001) and efficacy (β = 1.31, *p* = 0.005). For objective #3, as shown in Model 2 in Table 2, those who reused face masks (β = 2.14, *p* = 0.006) had more depressive symptoms. For objective #4, two health belief components were associated with depressive symptoms, namely severity (β = 0.88, *p* < 0.001) and cue (β = −0.57, *p* = 0.002).

**Table 2:** General linear model of face mask use and depressive symptoms.

	**Model 1 (*****N*** **= 350)**		**Model 2 (*****N*** **= 350)**	
	**Face mask use**		**Depressive symptoms**	
	**B (SE)**	***p*-value**	**B (SE)**	***p*-value**
Age	0.07 (0.08)	0.363	−0.05 (0.09)	0.550
Sex		0.054		0.756
Male	1.05 (0.54)		0.20 (0.63)	
Female	Ref		Ref	
Education level		0.175		0.712
Elementary or below	2.16 (1.39)	0.121	1.31 (1.61)	0.415
High school	0.66 (0.52)	0.209	0.14 (0.61)	0.815
University or higher	Ref		Ref	
No. of household members	0.07 (0.18)	0.686	−0.25 (0.21)	0.224
Depressive symptoms	−0.02 (0.47)	0.670	NA	NA
**Health beliefs**				
Susceptibility	−0.10 (0.28)	0.722	−0.02 (0.33)	0.956
Severity	0.63 (0.19)	0.001^*^	0.88 (0.21)	<0.001^*^
Cue to action	0.17 (0.16)	0.289	−0.57 (0.18)	0.002^*^
Knowledge	−0.09 (0.24)	0.724	−0.37 (0.28)	0.192
Efficacy	1.31 (0.46)	0.005^*^	−0.82 (0.53)	0.124
Face mask reuse		0.095		0.006^*^
Yes	1.126 (0.67)		2.14 (0.77)	
No	Ref		Ref	

* *Statistically significant at p < 0.05. B, beta; SE, standard error; NA, not applicable; Ref, reference*.

For objective #5, as shown in Table 3, Model 3 was applied separately to subjects with health beliefs pertaining to stronger (*n* = 148) and weaker (*n* = 207) beliefs in disease severity. Mask reuse was significantly associated with depression in the group with a stronger belief in disease severity (β = 3.02, *p* = 0.025), but not in the group with weaker perceptions of severity. As shown in Table 4, Model 4 was applied separately to subjects with high (*n* = 148) and low (*n* = 206) health belief scores in cues to preventive measures. Mask reuse was significantly associated with depression only in the group with poorer cues to preventive measures (β = 3.83, *p* = 0.004), but not in the group with better cues to preventive measures.

**Table 3:** Moderation effect of perceived severity on face mask reuse and depressive symptoms.

	**Model 3**			
	**Depressive symptoms in weaker belief in perceived severity^∧^**		**Depressive symptoms in stronger belief in perceived severity^∧^**	
	**(*****n*** **= 207)**		**(*****n*** **= 148)**	
	**B (SE)**	***p*-value**	**B (SE)**	***p*-value**
Age	−0.11 (0.12)	0.358	−0.17 (0.16)	0.279
Sex		0.666		0.391
Male	−0.33 (0.77)		0.97 (1.12)	
Female	Ref		Ref	
Education level		0.923		0.114
Elementary or below	−0.79 (3.00)	0.793	3.33 (2.16)	0.125
High school	−0.24 (0.74)	0.745	1.83 (1.05)	0.084
University or higher	Ref		Ref	
No. of household members	−0.01 (0.23)	0.956	−0.72 (0.41)	0.078
Face mask reuse		0.146		0.025^*^
Yes	1.37 (0.94)		3.02 (1.33)	
No	Ref		Ref	

* *Statistically significant at p < 0.05*.

∧ *high perceived severity = the subscale score for severity ≥ median score (6), low perceived severity = the subscale score for severity < median. B, beta; SE, standard error; Ref, reference*.

**Table 4:** Moderation effect of cues to preventive measures on face mask reuse and depressive symptoms.

	**Model 4**			
	**Depressive symptoms in poorer cues to preventive measures^∧^**		**Depressive symptoms in better cues to preventive measures^∧^**	
	**(*****n*** **= 206)**		**(*****n*** **= 148)**	
	**B (SE)**	***p*-value**	**B (SE)**	***p*-value**
Age	−0.03 (0.16)	0.839	−0.14 (0.12)	0.248
Sex		0.630		0.273
Male	−0.53 (1.10)		0.88 (0.80)	
Female	Ref		Ref	
Education level		0.195		0.649
Elementary or below	4.14 (2.77)	0.138	1.64 (2.03)	0.420
High school	1.29 (1.03)	0.213	0.45 (0.77)	0.564
University or higher	Ref		Ref	
No. of household members	−0.03 (0.31)	0.935	−0.45 (0.27)	0.102
Face mask reuse		0.004^*^		0.367
Yes	3.83 (1.31)		0.89 (0.98)	
No	Ref		Ref	

* *Statistically significant at p < 0.05*.

∧ *high cue to preventive measures = the subscale score for cue to action ≥ median score (14.5), low cue to preventive measures = the subscale score for sue to action < median. B, beta; SE, standard error; Ref, reference*.

## Discussion

To the best of our knowledge, this is the first study to report associations among depressive symptoms, the wearing of face masks, and health beliefs among the older population during the COVID-19 pandemic. It is important to understand the relationships among these variables in older people because the findings can inform the formulation of interventions and policies to promote mask wearing and reduce depressive symptoms during this pandemic.

In Hong Kong, depressive symptoms have been more severe among older people during the COVID-19 pandemic than before the pandemic period. In a study conducted in Hong Kong during the period between October 2010 and January 2012, the prevalence of depression as measured by the PHQ9 score above 9 was 9.8%, with a mean score of 3.57 in people aged 65 and older (35). This study showed that 25.6% of the subjects experienced depressive symptoms, with a mean PHQ9 score of 6.59, which is much higher than in the pre-pandemic period, although findings here might have been influenced by the nature of convenience sample in this cross-sectional study. A similar finding of a higher prevalence of depression (16.5%) in younger people aged 16–60 years during the COVID-19 pandemic has also been observed in Chinese communities (36). Another study showed that COVID-19 risk perception also increased the depressive symptoms in younger people (37). These findings might suggest that COVID-19 has intensified depressive symptoms among older people. The present study recommends that mental health services be proactively provided to older people during the current pandemic. Further studies should also be conducted to examine whether depression is more severe among older people in other countries.

Older people whose health beliefs allow them to perceive COVID-19 as serious and the wearing of face masks as an efficacious preventive measure use face masks more often. This supports the argument that the promotion of health beliefs is an important strategy to encourage face mask wearing among older people (38), although other factors may also possibly affect the wearing of face masks beyond health beliefs (e.g., access to face masks). During the shortage of face mask supply, access to face masks in individuals may vary because of older people's financial resources. Therefore, health education strategies should focus on highlighting the severity of the disease and promoting the efficacy of face mask use among older people as a public health policy in order to encourage older people to wear face masks during the current pandemic. Future studies should examine whether the effect of health beliefs on face mask wearing is independent of other factors (e.g., access to face masks).

Another important finding was that depressive symptoms were more clearly manifested among older people who: (a) reused face masks, (b) had a stronger belief in disease severity, and (c) did not have adequate cues to preventive measures. Disposable face masks are the most common type of face masks in Hong Kong and used to prevent respiratory infection by using multiple layers of filters made of non-woven fabrics that were designed for single use, while reusable face masks refer to face masks made of washable fiber micro-porous filters for repeated use (39). Reusing disposable face masks is known to be a suboptimal but necessary alternative when there is a shortage of face masks, but it has been common during the COVID-19 pandemic (40). Therefore, the reuse of disposable face masks is a reliable indicator of a shortage of face mask supply, limited access, limited affordability, and inadequate health information. A plausible reason for the reuse of face masks being associated with depression is that these older persons did not have an adequate supply of and access to face masks or limited knowledge of the efficacy of face mask wearing. Further analyses in this study showed that the reuse of face masks was only associated with depressive symptoms in the sub-groups of older people with a strong belief in disease severity and inadequate cues to preventive measures. These observations could be explained by older people experiencing greater depressive symptoms if they know that COVID-19 is severe, but they do not have appropriate cues to preventive measures (e.g., face masks) to protect them from contracting COVID-19. Having said that, some other factors which were not included in the analysis may possibly confound these observed associations, such as socioeconomic status.

These two health belief factors also moderated the effect of face mask reuse on depressive symptoms among older people. In addition to participants' differences in their health beliefs pertaining to the pandemic, there might have been other underlying factors that might have confounded our findings. Face mask reuse might also be the result of lacking access to face mask supply because of various reasons, such as socioeconomic factors. Further studies should examine these relationships. We recommend that mental health support and cues to preventive measures should be offered to older people alongside COVID-19 prevention-related health education, particularly those focusing on the severity of the disease. Policymakers should work closely to increase the supply of and access to face masks. Researchers should investigate whether alternatives such as wearing reusable face masks, face shields, or decontaminating face masks are effective when face masks are in short supply (41, 42).

This study has several limitations. As face-to-face interviews were prohibited during the pandemic, an online survey was used to collect data widely and quickly. Convenience sampling and employing online methodological strategies for the survey may have induced selection bias in this study. Unfamiliarity with technology and lack of access to technology among some older people might have excluded them from this survey. These might have posed a risk that older adults who were included in this study were more financially resourceful and possibly received more formal education than those who were not included. Hence, the sample in this study might not be the most accurate reflection of the reality in the greater population of community-dwelling older people. There are other possible confounders affecting depressive symptoms (e.g., comorbidities and anxiety) that were not adjusted for in this study. Therefore, this study could not conclude whether face mask reuse and health beliefs led to more depressive symptoms independent of underlying comorbidities and anxiety levels. The prevalence of depressive symptoms was based on relatively small sample size and convenience sampling. Therefore, the 95% CI was measured as 4.5, which was larger than that in another study (95% CI = 2.0) surveying for depression in the same population in Hong Kong (35). We are less confident that the prevalence of depression is accurate within a narrow confidence range. Caution should be exercised when the prevalence of depression is extrapolated beyond the examined group. We adopted a simple statistical analysis to test the moderation effect of health beliefs without testing the significance of the effect because of the small sample size. Finally, this study focused only on the use of disposable face masks. The use of reusable face masks was not examined because there was limited evidence of the effectiveness of reusable face masks during the study period.

To conclude, health beliefs regarding perceived severity and efficacy contributed to more frequent face mask use, which is unrelated to depressive symptoms. Older people with a stronger belief in disease severity and with poorer cues to preventive measures, as well as those who reuse disposable face masks, are more likely to experience depressive symptoms. A moderation effect of health beliefs (i.e., disease severity and cues to preventive measures) on face mask reuse and depression was observed. Mental health support is therefore as important as health education for promoting health beliefs toward prevention against COVID-19.

## Data Availability Statement

The raw data supporting the conclusions of this article will be made available by the authors, without undue reservation.

## Ethics Statement

The studies involving human participants were reviewed and approved by Human Subjects Ethics Subcommittee of The Hong Kong Polytechnic University (Reference number: HSEARS20200227002-01). The participants provided their written informed consent to participate in this study.

## Author Contributions

RK, PL, DC, and SL discussed and formulated the research idea. SL coordinated the data collection. PL conducted the statistical analysis. RK wrote the first draft of the manuscript. PL, DC, and SL commented on and revised the manuscript. All authors reviewed and approved the final version of the manuscript.

## Conflict of Interest

The authors declare that the research was conducted in the absence of any commercial or financial relationships that could be construed as a potential conflict of interest.

## References

[1] World Health Organization Coronavirus Disease (COVID-19) Weekly Epidemiological Update. (2020). Available online at: https://www.who.int/publications/m/item/weekly-epidemiological-update (accessed October 27, 2020).

[2] Zhou F Yu T Du R Fan G Liu Y Liu Z. Clinical course and risk factors for mortality of adult inpatients with COVID-19 in Wuhan, China: a retrospective cohort study. Lancet. (2020) 395:1054–62. 10.1016/S0140-6736(20)30566-3 32171076 PMC7270627

[3] Bressington DT Cheung TCC Lam SC Suen LKP Fong TKH Ho HSW. Association between depression, health beliefs, and face mask use during the COVID-19 pandemic. Front Psych. (2020) 11:571179. 10.3389/fpsyt.2020.571179 33192697 PMC7642487

[4] The Government of the Hong Kong SAR Coronavirus Disease (COVID-19) in HK. (2020). https://www.coronavirus.gov.hk/eng/index.html (accessed October 25, 2020).

[5] Wong SY Kwok KO Chan FKJC. What can countries learn from Hong Kong's response to the COVID-19 pandemic? CMAJ. (2020) 192:E511–5. 10.1503/cmaj.200563 32332040 PMC7234274

[6] Cheng VCC Wong SC Chuang VWM So SYC Chen JHK Sridhar S. The role of community-wide wearing of face mask for control of coronavirus disease 2019 (COVID-19) epidemic due to SARS-CoV-2. J Infect. (2020) 81:107–14. 10.1016/j.jinf.2020.04.024 32335167 PMC7177146

[7] Kobayashi LC Wardle J Wolf MS von Wagner C. Aging and functional health literacy: a systematic review and meta-analysis. J Gerontol B. (2014) 71:445–57. 10.1093/geronb/gbu161 25504637 PMC4834761

[8] Nguyen HC Nguyen MH Do BN Tran CQ Nguyen TT Pham KM. People with suspected COVID-19 symptoms were more likely depressed and had lower health-related quality of life: the potential benefit of health literacy. J Clin Med. (2020) 9:965. 10.3390/jcm9040965 32244415 PMC7231234

[9] Lam SC Suen LKP Cheung TCC. Global risk to the community and clinical setting: flocking of fake masks and protective gears during the COVID-19 pandemic. Am J Infect Control. (2020) 48:964–5. 10.1016/j.ajic.2020.05.008 32405127 PMC7219383

[10] Ni MY Yao XI Leung KSM Yau C Leung CMC Lun P. Depression and post-traumatic stress during major social unrest in Hong Kong: a 10-year prospective cohort study. Lancet. (2020) 395:273–84. 10.1016/S0140-6736(19)33160-5 31928765

[11] Rodda J Walker Z Carter J. Depression in older adults. BMJ. (2011) 343:d5219. 10.1136/bmj.d5219 21957206

[12] Colasanti V Marianetti M Micacchi F Amabile GA Mina C. Tests for the evaluation of depression in the elderly: a systematic review. Arch Geront Geriatr. (2010) 50:227–30. 10.1016/j.archger.2009.04.001 19414202

[13] Qiu J Shen B Zhao M Wang Z Xie B Xu Y. A nationwide survey of psychological distress among Chinese people in the COVID-19 epidemic: implications and policy recommendations. Gen Psychiatry. (2020) 33:e100213. 10.1136/gpsych-2020-100213 32215365 PMC7061893

[14] Girdhar R Srivastava V Sethi S Managing mental health issues among elderly during COVID-19 pandemic. J Geriat Care Res. (2020) 7:29–32. 10.1016/j.cmrp.2020.07.016

[15] Clayborne ZM Colman I. Associations between depression and health behaviour change: findings from 8 cycles of the Canadian Community Health Survey. Can J Psychiatry. (2018) 64:30–8. 10.1177/0706743718772523 29734818 PMC6364138

[16] Feng S Shen C Xia N Song W Fan M Cowling BJ. Rational use of face masks in the COVID-19 pandemic. Lancet Respir Med. (2020) 8:P434–36. 10.1016/S2213-2600(20)30134-X 32203710 PMC7118603

[17] World Health Organization Advice on the Use of Masks in the Context of COVID-19: Interim Guidance. World Health Organization (2020). Available online at: https://apps.who.int/iris/handle/10665/332293 (accessed June 5, 2020).

[18] Bai Y Yao L Wei T Tian F Jin D-Y Chen L. Presumed asymptomatic carrier transmission of COVID-19. JAMA. (2020) 323:1406–7. 10.1001/jama.2020.2565 32083643 PMC7042844

[19] Greenhalgh T Schmid MB Czypionka T Bassler D Gruer L. Face masks for the public during the covid-19 crisis. BMJ. (2020) 369:m1435. 10.1136/bmj.m1435 32273267

[20] Cheng KK Lam TH Leung CC. Wearing face masks in the community during the COVID-19 pandemic: altruism and solidarity. Lancet [Preprint]. (2020). 10.1016/S0140-6736(20)30918-1 PMC7162638 32305074

[21] Kipgen N Stop Discrimination and Fight the Virus. (2020). Available online at: https://www.thestatesman.com/opinion/stop-discrimination-fight-virus-1502871009.html (accessed June 1, 2020).

[22] Centre for Health Protection Guidelines on Prevention of COVID-19 for the General Public. (2020). Available online at: https://www.chp.gov.hk/files/pdf/nid_guideline_general_public_en.pdf (accessed December 8, 2020).

[23] Centre for Health Protection Guidelines for Residential Care Homes for the Elderly or Persons with Disabilities for the Prevention of COVID-19 (Interim). (2020). Available online at: https://www.chp.gov.hk/files/pdf/advice_to_rche_rchd_on_prevention_of_nid_eng.pdf (accessed December 8, 2020).

[24] Champion VL Skinner CS The Health Belief Model. In: Glanz K Rimer BK Viswanath K, editors. Health Behavior and Health Education. Theory, Research, and Practice. San Francisco, CA: Jossey-Bass (2008). p. 45–65.

[25] Lam SC Arora T Grey I Suen LKP Huang EY-z Li D. Perceived risk and protection from infection and depressive symptoms among healthcare workers in Mainland China and Hong Kong During COVID-19. Front Psychiatr. (2020) 11:686. 10.3389/fpsyt.2020.00686 32765321 PMC7378321

[26] Mukhtar S. Mental health and emotional impact of COVID-19: applying health belief model for medical staff to general public of Pakistan. Brain Behav Immun. (2020) 87:28–9. 10.1016/j.bbi.2020.04.012 32283289 PMC7151322

[27] Connell CM Storandt M Lichty W Impact of health belief and diabetes-specific psychosocial context variables on self-care behavior, metabolic control, and depression of older adults with diabetes. Behav, Health, Aging. (1990) 1:183–96.

[28] Kotrlik J Higgins C Organizational research: determining appropriate sample size in survey research appropriate sample size in survey research. Inf Technol Learn Perf J. (2001) 19:43.

[29] Ahmed MZ Ahmed O Aibao Z Hanbin S Siyu L Ahmad A. Epidemic of COVID-19 in China and associated psychological problems. Asian J Psychiatry. (2020) 51:102092. 10.1016/j.ajp.2020.102092 32315963 PMC7194662

[30] Kroenke K Spitzer RL Williams JB. The PHQ-9: validity of a brief depression severity measure. J Gen Inter Med. (2001) 16:606–13. 10.1046/j.1525-1497.2001.016009606.x 11556941 PMC1495268

[31] Chen S Chiu H Xu B Ma Y Jin T Wu M. Reliability and validity of the PHQ-9 for screening late-life depression in Chinese primary care. Int J Geriatr Psychiatry. (2010) 25:1127–33. 10.1002/gps.2442 20029795

[32] Tang CS-k Wong C-y. Factors influencing the wearing of facemasks to prevent the severe acute respiratory syndrome among adult Chinese in Hong Kong. Prev Med. (2004) 39:1187–93. 10.1016/j.ypmed.2004.04.032 15539054 PMC7133369

[33] Lam SC Chong ACY Chung JYS Lam MY Chan LM Shum CY Methodological study on the evaluation of face mask use scale among public adult: cross-language and psychometric testing. Korean J Adult Nurs. (2020) 32:46–56. 10.7475/kjan.2020.32.1.46

[34] The Government of Hong Kong Special Administrative Region Hong Kong 2016 Population By-census - Thematic Report: Older Persons. Hong Kong: Department CaS (2016).

[35] Chin WY Chan KTY Lam CLK Wong SYS Fong DYT Lo YYC. Detection and management of depression in adult primary care patients in Hong Kong: a cross-sectional survey conducted by a primary care practice-based research network. BMC Fam Pract. (2014) 15:30. 10.1186/1471-2296-15-30 24521526 PMC3937039

[36] Wang C Pan R Wan X Tan Y Xu L Ho CS. Immediate psychological responses and associated factors during the initial stage of the 2019 coronavirus disease (COVID-19) epidemic among the general population in China. Int J Env Res Pub Health. (2020) 17:1729. 10.3390/ijerph17051729 32155789 PMC7084952

[37] Okruszek Ł Aniszewska-Stańczuk A Piejka A Wiśniewska M Zurek K. Safe but lonely? Loneliness, anxiety, and depression symptoms and COVID-19. Front Psychol. (2020) 11:579181. 10.3389/fpsyg.2020.579181 33343454 PMC7747668

[38] Sim SW Moey KSP Tan NC. The use of facemasks to prevent respiratory infection: a literature review in the context of the health belief model. Singapore Med J. (2014) 55:160–7. 10.11622/smedj.2014037 24664384 PMC4293989

[39] Lee K-P Yip J Kan C-W Chiou J-C Yung K-F. Reusable face masks as alternative for disposable medical masks: factors that affect their wear-comfort. Int J Environ Res Public Health. (2020) 17:6623. 10.3390/ijerph17186623 32932918 PMC7558362

[40] Purens AGM. Facemask alternatives in veterinary medicine in the context of COVID-19 shortages. Front Vet Sci. (2020) 7:561. 10.3389/fvets.2020.00561 33062649 PMC7482367

[41] Farsi D Mofidi M Mahshidfar B Hafezimoghadam P Consider the options; can decontamination and reuse be the answer to N95 respirator shortage in COVID-19 pandemic? Adv J Emerg Med. (2020) 4:e41 10.22114/ajem.v4i2s.378

[42] Zorko DJ Gertsman S O'Hearn K Timmerman N Ambu-Ali N Dinh T. Decontamination interventions for the reuse of surgical mask personal protective equipment: A systematic review. J Hosp Infect. (2020) 106:283–94. 10.1016/j.jhin.2020.07.007 32653432 PMC7347478

